# Supplementary material for: Long-term performance assessment of fully automatic biomedical glottis segmentation at the point of care
Source: PLoS One. 2022 Sep 21;17(9):e0266989. doi: 10.1371/journal.pone.0266989 (PMC9491538; doi:10.1371/journal.pone.0266989)
Supplement: S1 File — (PDF) [file pone.0266989.s001.pdf]

Supporting information

Long-term performance assessment of fully automatic biomedical glottis segmentation at the point of care

René Groh, Stephan Dürr, Anne Schützenberger, Marion Semmler, and Andreas M. Kist

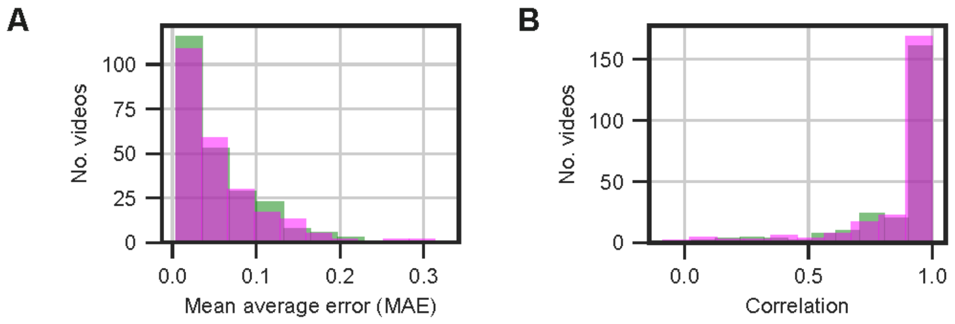

Supplementary Figure 1. GAWs are highly correlated across compression methods. A: Distribution of mean absolute error (MAE) across compression modes segmented w/ ROI (green) or w/o ROI (magenta). **B**: Distribution of the Pearson's correlation coefficient across compression modes segmented w/ ROI (green) or w/o ROI (magenta).

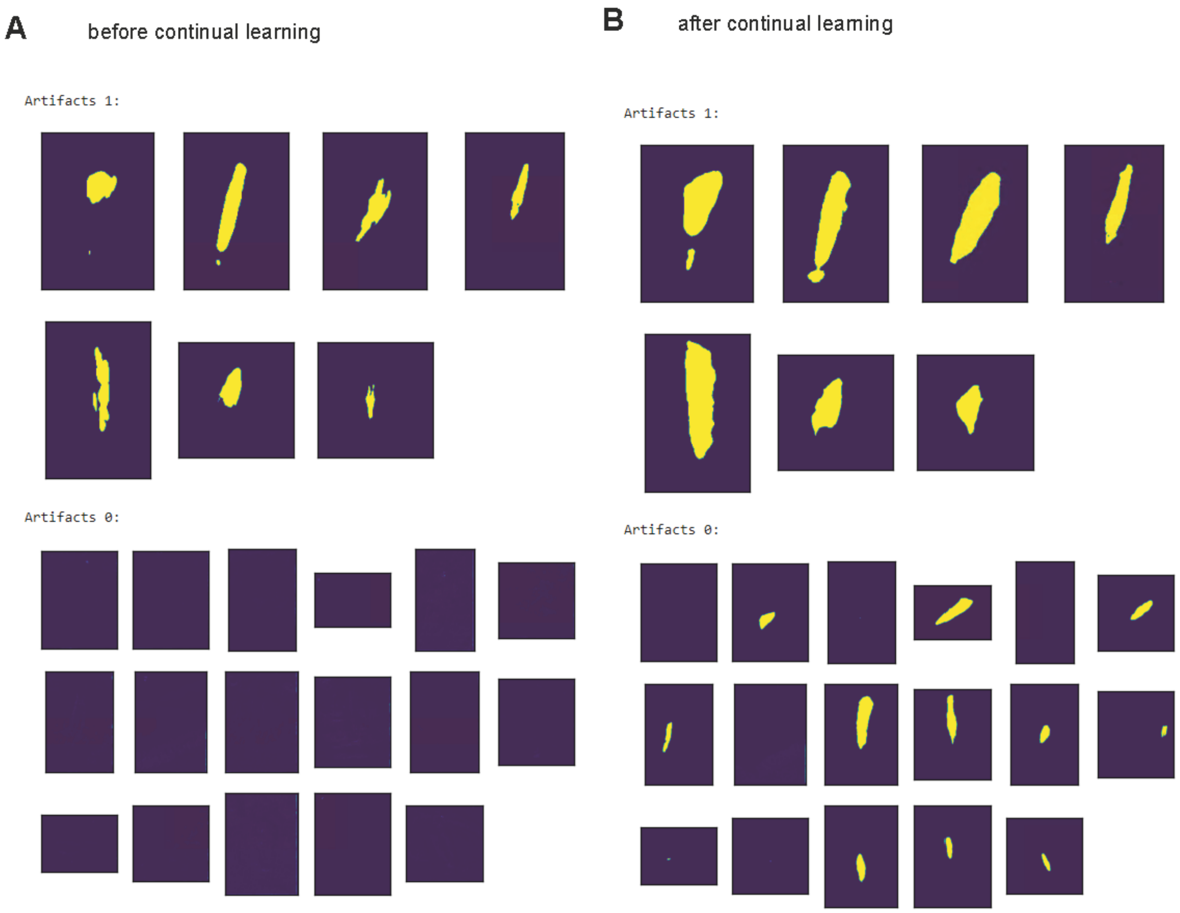

Supplementary Figure 2. Continual learning has an effect on erroneous segmentations (upper panels, rated as "1") and on failed segmentations (lower panels, rated as "0").

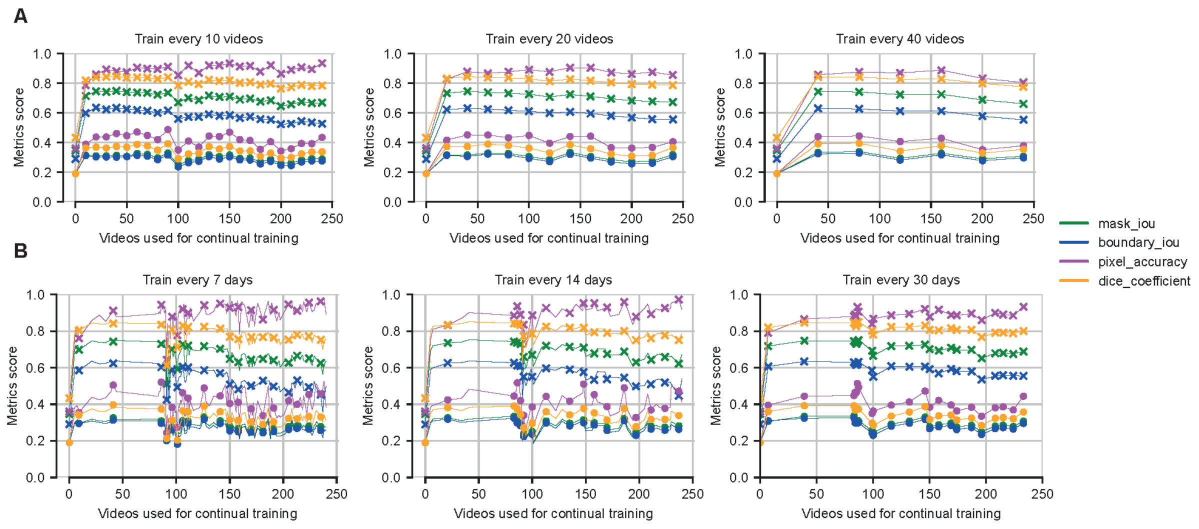

Supplementary Figure 3. Various metrics show similar behavior as Mask-IoU.
A. Measurement of continual learning performance with a fixed amount of videos and different metrics for videos with artifacts depending on the rating (dots: 0=large artifacts, failed segmentations, crosses: 1=small artifacts, erroneous segmentations. **B.** Same as panel A, but continual learning with fixed time intervals.

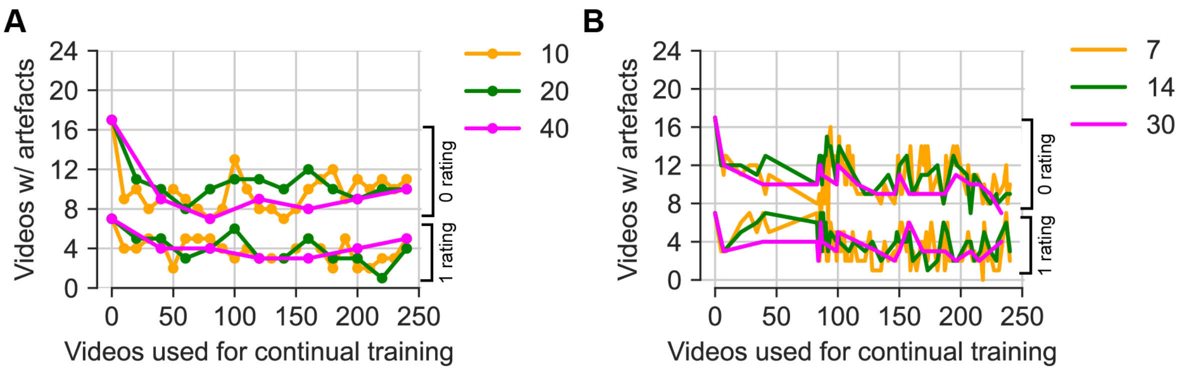

Supplementary Figure 4. artifacts decay with continual learning. A. Videos with artifacts according to rating (0=large artifacts, failed segmentations, 1=small artifacts, erroneous segmentations) for continual learning with a fixed amount of videos, namely 10 (yellow), 20 (green) or 40 (magenta). **B**. Same as panel A, but with fixed time interval of 7 (yellow), 14 (green) or 30 (magenta) days.

	**Artefacts Rating 0**		**Artefacts Rating 1**	
	# Artefacts	IoU	# Artefacts	IoU
Custom UNet	14/17	0.21	4/7	0.47
EfficientNetB0	10/17	0.35	1/7	0.57
ResNet50	8/17	0.45	2/7	0.54
Reference	17/17	0.19	7/7	0.34

Supplementary Table 1. Larger pre-trained DNNs perform slightly better in glottal segmentation before fine-tuning our model. Number of artifacts and Intersection over Union (IoU) for several deep neural networks.
